# *Paraboremia selaginellae* enhances *Salvia miltiorrhiza* growth and cadmium tolerance via modulating root architecture and cadmium speciation in contaminated environments

**DOI:** 10.3389/fpls.2025.1540126

**Published:** 2025-04-01

**Authors:** Yuli Lin, Zhenzhou Wang, Wenjing Chen, Yunfeng Liu, Xia Li, Hongliang Tang, Xueli He

**Affiliations:** ^1^ College of Life Sciences, Hebei University, Baoding, China; ^2^ Key Laboratory of Microbial Diversity Research and Application of Hebei Province, Baoding, China

**Keywords:** plant performance, dark septate endophytes, chemical form of Cd, soil factor, *Salvia miltiorrhiza*, Cd contaminated environment

## Abstract

**Introduction:**

Dark septate endophytes (DSEs) commonly colonize plant roots in heavy metal-polluted habitats, but little is known about the potential mechanisms driving medicinal plants to adapt to heavy metal-polluted environments. Here, we investigated the growth of *Paraboeremia selaginellae* strains and their enhancing of cadmium (Cd) tolerance and growth in *Salvia miltiorrhiza* in Cd-contaminated soil.

**Methods:**

First, we tested the Cd resistance of *P. selaginellae* by *in vitro* culture. Then, we studied the performance of *S. miltiorrhiza* inoculated with *P. selaginellae* in Cd-contaminated soil.

**Results:**

It was found that *P. selaginellae* possessed a capacity to accumulate Cd in its mycelium. The Cd-contaminated environment increased the content of melanin and osmotic substances and reduced the proportion of the highly toxic chemical form of Cd in the mycelium of *P. selaginellae*. In Cd-contaminated soil, *P. selaginellae* effectively colonized plant roots and improved soil nutrients and the growth of *S. miltiorrhiza*. The *P. selaginellae*-inoculated treatment significantly increased the contents of effective nitrogen (37.74%), urease (31.55%), and alkaline phosphatase (29.82%) in 10 mg Cd/kg soil, compared with the non-inoculated treatment. More importantly, *P. selaginellae* inoculation increased root biomass for 5 and 10 mg Cd/kg soil by 42.35% and 30.21%, respectively, in comparison to non-inoculated control. Inoculation with *P. selaginellae* reduced the proportion of the highly toxic chemical form of soil Cd, and reduced the accumulation of Cd in plants, especially in the roots.

**Discussion:**

These results indicated that DSEs have a positive influence on the growth and Cd tolerance of *S. miltiorrhiza*, and reduce the biological toxicity of soil Cd. Therefore, exploitation and utilization of DSEs resources might be a new method for improving the cultivation of medicinal plants and soil microenvironment in heavy metal-contaminated areas.

## 1 Introduction

Heavy metal (HM) pollution is a very serious environmental problem, which not only seriously affects plant growth and agricultural production but also seriously threatens and destroys biodiversity and soil quality (Gallego et al., 2012; Hu et al., 2014). Therefore, preventing and controlling heavy metal pollution in soil has great significance for agricultural production and soil quality improvement. Studies have shown that microbial remediation is a high-efficiency and low-cost ecological restoration technology. In particular, plant symbiotic microorganisms can have the dual advantages of plant remediation and microbial remediation, improving the efficiency of heavy metal-contaminated soil remediation, and show good application potential (Cozzolino et al., 2016; Zhu et al., 2021).

Generally colonizing live plant roots without evident detrimental consequences, dark septate endophytes (DSEs) are distinguished by the presence of melanized microsclerotia and dark septate hyphae (Jumpponen and Trappe, 1998). DSEs are widely distributed in various ecological environments, with especially high colonization rates in stress environments such as drought and heavy metal pollution (Zhan et al., 2015). Previous studies have shown that most DSEs can adsorb heavy metals and show strong tolerance to HM stress. They could also degrade, move, or fix pollutants in the soil, reduce plant toxicity, promote plant growth, and improve plant tolerance to heavy metals (Rahman et al., 2020; Shah and Daverey, 2020; Su et al., 2021). DSEs’ hyphae are rich in melanin, which can enhance the rigidity of the cell wall structure and improve fungal tolerance to environmental stresses such as heavy metals and drought (Berthelot et al., 2017b; Eisenman and Casadevall, 2011). After DSEs colonize plant roots, they form a mycelial network system in the soil, which can promote root water and nutrient absorption, regulate plant metabolic activities, and improve plant resistance to HMs (Ban et al., 2017). In addition, DSEs not only affect the host plant but also decompose soil organic compounds, convert soil organic nutrients into effective forms, and affect soil carbon, nitrogen, and phosphorus cycles (Baum et al., 2018; Challacombe et al., 2019). Therefore, DSEs are considered a beneficial microbial resource with great potential for application in plant growth and bioremediation technology (Aslam et al., 2021; Salducci et al., 2019), and a comprehensive understanding of the ecological adaptation and tolerance mechanisms of DSEs, as well as DSEs plant symbiosis, can lay the foundation for fully realizing their application potential.

As an important component of natural ecosystems, soil heavy metal pollution has attracted much attention due to its damage to soil ecological functions and the decline in agricultural product quality, especially cadmium (Cd) pollution in soil, which was particularly prominent (Ignatova et al., 2021; Ma et al., 2022). After Cd enters the soil, due to its poor mobility, long residence time in the soil, and various chemical forms, it easily causes soil fertility degradation and an imbalance of soil biological processes and microbial community structure, and once it is absorbed by organisms and enters the food chain, it seriously threatens human life and health (Xue et al., 2022). Medicinal plants have a wide range of uses worldwide, but Cd pollution in cultivated soil can easily endanger the safety of medicinal herbs (Fan et al., 2023). Therefore, the remediation of contaminated soil remains an important issue for soil environment management and improving the quality of medicinal plants. However, the current understanding of the interactions between DSEs and medicinal plants in Cd-contaminated environments and their contributions to alleviating Cd stress remains limited.

*Salvia miltiorrhiza* Bge. (Labiaceae), a common medicinal plant, is used to treat a wide range of illnesses, including tumors, decreasing blood pressure and cholesterol, treating and preventing atherosclerosis, and increasing blood circulation and relieving blood stasis (Wu et al., 2023a). The active ingredients are divided into water-soluble and fat-soluble components, among which salvianolic acid B is an important water-soluble component, and the fat-soluble components mainly include cryptotanshinone, tanshinone I, and tanshinone IIA (Shan et al., 2024; Xiao et al., 2020). Soil Cd pollution poses a significant threat to the growth of *Salvia miltiorrhiza*. This plant is highly susceptible to accumulating excessive amounts of Cd from contaminated soil, which can subsequently compromise the quality of *S. miltiorrhiza* (Wei et al., 2020). Previous studies have shown that the application of mercapto-based palygorskites promoted the growth of *S. miltiorrhiza* and inhibited the accumulation of cadmium and lead by regulating soil microecology (Wu et al., 2023b). Our previous studies found that inoculation of DSEs in sterilized soil significantly increased the biomass and Cd tolerance of plants such as *Medicago sativa* and *Ammopiptanthus mongolicus* under Cd stress (Hou et al., 2020). In addition, DSE inoculation has been shown to mitigate the negative impacts of Cd stress on the growth of *Astragalus mongholicus* (Li et al., 2024). The aim of this study was to reveal the effects and potential mechanisms of DSE inoculation in medicinal plants in Cd-contaminated soil. First, Cd stress tolerance tests were conducted on the DSE strain *Paraboremia selaginellae* stored in the laboratory. Subsequently, pot experiments were conducted to investigate the effects of inoculation with this strain in different Cd-contaminated soils on the growth of *S. miltiorrhiza*, in order to answer the following questions: (1) Does *P. selaginellae* exhibit high tolerance to a Cd-contaminated environment *in vitro*? (2) Does inoculation with *P. selaginellae* reduce the Cd content in *S. miltiorrhiza*, especially in the roots? (3) Does inoculation with *P. selaginellae* affect the forms of Cd occurrence in soil? If yes, (4) Does inoculation with *P. selaginellae* promote the growth of *S. miltiorrhiza* in Cd-contaminated soil?

## 2 Materials and methods

### 2.1 Fungi and plant materials

The DSE strain used in the experiment was isolated from the roots of *Astragalus membranaceus* that grew naturally in an environment affected by multiple heavy metals, including Cd, chromium (Cr), and copper (Cu), and was preserved in the Strain Collection Center of the Mycorrhizal Biology Laboratory of Hebei University.

Approximately 50 mg of fresh DSE hyphae was collected, and genomic DNA extraction was performed using a DNA extraction kit (SolarBio, Beijing, China). The PCR primers used were ITS4 and ITS5. The PCR products were then purified and sequenced. Sequence alignment was performed using ClustalX (v.1.81) software. A maximum likelihood phylogenetic tree was constructed using MEGA6.0. The DNA sequences were deposited in GenBank with accession numbers MT723848-MT723861. The DSE strains were phylogenetically identified as *Paraboeremia selaginellae* (PS-2) (Han, 2022). The strain was cultured on Petri dishes with potato dextrose agar (PDA) medium at 27°C in the dark for 14 d for subsequent experiments.

*Salvia miltiorrhiza* seeds were obtained from the Hebei Anguo Medicinal Plant Base, and all seeds were stored at 4°C until germination.

### 2.2 DSE tolerance to Cd-contaminated environment *in vitro*

DSE solid culture: Cadmium chloride (CdCl_2_·2.5H_2_O) was added to Modified Melin-Norkrans Medium (MMN) solid culture medium to prepare the Cd stress culture, with Cd concentrations of 0, 20, 40, and 60 mg Cd/L in different treatments of MMN medium. The medium was sealed with sterile film and sterilized with high-pressure steam (121°C, 15 min) for use. Under sterile conditions, 5 mm fungal disks were taken from the PS-2 colonies, activated for 14 d, and inoculated into MMN solid medium centers with different Cd concentrations. Each treatment was repeated three times, and 2 cm × 2 cm coverslips were inserted around the fungal disks. The culture was inverted in a constant temperature incubator at 27°C in the dark for 14 d, and the microscopic characteristics were observed with an optical microscope.

DSE liquid culture: Cd chloride (CdCl_2_·2.5H_2_O) was added to MMN liquid medium to prepare th Cd stress culture, with Cd concentrations of 0, 20, 40, and 60 mg Cd/L in different treatments of MMN medium, and the medium was sealed with sterile film and sterilized with high-pressure steam (121°C, 15 min) for use. Three 5 mm fungal disks were taken from the PS-2 colony, activated for 14 d, and inoculated into MMN liquid medium with different concentrations. Each treatment was repeated three times and cultured in a constant temperature shaker (27°C, 150 r/min) for 20 d. After being cultivated in liquid medium, PS-2 mycelium was first congregated by suction filter, and the fresh mycelium was randomly separated into two parts: one part was weighed and dried to constant weight at 80°C to measure moisture content, Cd content, and soluble sugar content, and the other part was used to determine the content of malondialdehyde (MDA), soluble protein, melanin, and the chemical form of Cd. The dry weight of the two parts was the total biomass of PS-2.

#### 2.2.1 Determination of the physiological indexes of DSE strains

The thiobarbituric acid (TBA) method was used to evaluate the MDA level of the mycelium (Jiao et al., 2020). Fresh mycelium 0.2 g was weighed into a centrifuge tube, 10% trichloroacetic acid was then added. After grinding using a high-throughput tissue grinder, the material was homogenized and then centrifuged at 10,000 × g for 10 min. A glass test tube was filled with supernatant and 0.5% TBA solution and the mixture was submerged in a distilled water bath set at 100°C. Using a spectrophotometer, the absorbance was determined at 450, 532, and 600 nm.

The Coomassie Brilliant Blue method was used to measure the mycelium’s soluble protein concentration (Hou et al., 2007). A centrifuge tube was filled with 0.2 g of fresh mycelium and 50 mM phosphate buffer. Following grinding with a high-throughput tissue grinder, the homogenate was centrifuged at 10,000×g. Using a spectrophotometer, the absorbance at 595 nm was measured after the mixture containing supernatant, distilled water, and Coomassie Brilliant Blue was left to stand for 5 min.

The anthrone colorimetric method was used to determine the mycelium’s soluble sugar concentration (Wang et al., 2024). Using a centrifuge tube, 0.05 g of dry mycelium was weighed out. This was extracted twice at 80°C using 80% ethanol (v/v); the supernatant was collected and combined, and a small amount of activated carbon was added. This mixture was then decolorized at 80°C for 30 minutes, 10 mL of deionized water was added, and after filtering, to extract the filtrate, 250 µL of the filtrate was mixed in 5 mL of anthracene solution and placed in a deionized water bath at 100°C for 10 min. Using a spectrophotometer, the absorbance was measured at 625 nm.

The extraction of hyphae melanin was carried out using the NaOH extraction method (Ellis and Griffiths, 1974). Thus, 0.05 g of fresh mycelium was weighed into a glass test tube, 1 M NaOH was added, and the mixture was incubated at 100 °C for 5 h. After cooling, the mycelium was filtered, and 7 M HCl (pH 2.0) was added. After precipitation extraction, it was washed, dissolved with 1M NaOH, and centrifuged at 10,000 × g. Using a spectrophotometer, the absorbance was measured at 459 nm.

#### 2.2.2 Determination of the Cd content and chemical forms of DSE strains

Dried mycelium (about 0.05 g) was digested in an HNO_3_/HClO_4_ (3:1) (v/v) digestion solution, and 0.2% HNO_3_ was used to dilute the mixture to 50 mL. An inductively coupled plasma optical emission spectrometer (ICP-OES, Beijing Ruiguang General Instrument Co., Ltd.) was used to calculate the mass fraction of Cd in DSE mycelium.

The chemical forms of Cd in the strain were extracted using the following specified solutions (Yang et al., 2000): (1) FE-Cd (80% ethanol, mainly extracting Cd in the form of inorganic salts and amino acid salts), (2) FW-Cd (deionized water, mainly extracting water-soluble complexes formed by Cd and organic acids), (3) FNaCl-Cd (1 M NaCl, mainly extracting Cd bound to proteins or pectins), (4) FHAc-Cd (2% acetic acid, mainly extracting insoluble cadmium phosphate), and (5) FHCl-Cd (0.6 M hydrochloric acid, mainly extracting Cd bound to oxalic acid). After being placed in a 25°C constant temperature incubator for 22 h, the supernatant was separated by centrifugation at 5000×g for 10 min, and 10 mL of the same extractant was added to the precipitate in the centrifuge tube. After being oscillated at 25°C for 2 h, the supernatant was centrifuged at 5000×g, and the process was repeated twice. The supernatants after three centrifugations were combined, and the residue was subjected to the next extraction. The final residue was the residue state (FR-Cd). All supernatants and residues were evaporated to constant weight at 70°C and digested with HNO_3_/HClO_4_ (3:1, v/v). After digestion, the volume was fixed to 50 mL, and the content of Cd in different forms was determined by ICP-OES.

### 2.3 Inoculation assay

This experiment used a two-factor random group design. Factor one was the DSE inoculation: inoculated with DSE and uninoculated with DSE (CK). Factor two was different Cd concentrations: 0, 5, and 10 mg Cd/kg contaminated soil. The test plant was *S. miltiorrhiza*, and each treatment was set up with four replicates and cultivated in non-sterile soil for 180 d.

The surface of the seeds of *S. miltiorrhiza* was sterilized with 70% ethanol and sodium hypochlorite for 180 s and 600 s, respectively. Finally, the sterilized seeds were rinsed with sterile deionized water and deposited on water agar medium (10 g/L agar) at a constant temperature of 27°C. All operations were performed under sterile conditions.

After pre-germination, the seedlings were placed in sterile pots with dimensions of 140 mm in the upper diameter, 950 mm in the lower diameter, and 115 mm in height; each pot held three seedlings. The pots were filled with 1300 g of non-sterile culture substrata, which was made up of river sand and farmland soil at a 2:1 (v/v) ratio. The culture substrata included 4.33 mg of organic carbon per gram, 2.8 µg of available nitrogen, 2.98 µg of available phosphorus, 108.27 µg of available potassium, 0.39 µg of Cd, and pH 7.47. DSE isolates were grown aseptically in Petri dishes using PDA culture medium to create DSE inocula. In order to inoculate *S. miltiorrhiza* seedlings with DSE, two 5 mm plugs that had been removed from the edge of an actively growing colony on culture medium were inserted at a distance of 1 cm from the roots. Plugs taken out of the medium without any fungus were used to inoculate the control treatments. Every inoculation procedure was carried out on a sterile bench. The potted seedlings were grown in a light incubator with a photoperiod of 10 h and a relative humidity of 60%. The daytime temperature was maintained at 27°C, and a nighttime temperature of 22°C was sustained.

The *S. miltiorrhiza* seedlings were treated with Cd stress after 30 d of development. To produce varied soil Cd pollution (5 and 10 mg Cd/kg soil), 50 mL of a CdCl_2_·2.5H_2_O solution of different concentrations was applied to the culture matrix of the flower pots with different treatments. The control treatment received 50 mL of deionized water (0 mg Cd/kg soil). A soil humidity recorder (L99-TWS-2, China) was used to measure the moisture content of the soil, and 180 d after sowing, the plants were harvested.

#### 2.3.1 Measurement of plant biomass and morphological traits

The height and leaf count of the *S. miltiorrhiza* plants were measured at the conclusion of the plant growth enhancement experiment. Each pot’s plant shoots and roots were plucked separately, and the roots’ surface-remaining soil was gently washed with deionized water. The root samples were scanned using an EPSON V800 scanner (Seiko Epson Inc., NKS, Tokyo, Japan) after being suspended in deionized water in a transparent tray for approximately 1 cm. The WinRHIZO image analysis system was used to examine the root morphology markers. Following scanning, the roots were gathered, and in order to calculate the plant biomass, some root and shoot samples were dried for more than 48 h at 70°C in an oven. The remaining fresh shoots and root samples were stored in an ultra-low temperature freezer at -80°C for future use.

#### 2.3.2 Determination of DSE colonization

Following a 0.5% (w/v) acidic magenta stain, fresh root segments (about 0.5 cm) were rinsed in 10% KOH solution (w/v), and they were then dissociated in a 100°C deionized water bath for 1 h and decolorized with lactic acid-glycerol. Under a light microscope, the DSE colonization structure and infection state were noted, and the DSE colonization rate was computed using the following formula (Phillips and Hayman, 1970):

Colonization rate (total, hyphae, and microsclerotia) (%) = number of infected root segments/total number of root segments.

#### 2.3.3 Measurement of plant physiological indexes

The apex fully developed leaf was used to assess the chlorophyll content of the leaves before the harvest (Spad-502, Konica Minolta Sensing, Inc., Osaka, Japan). The maximum absorbance values of Chl a and b at 650 nm were measured by this analyzer.

The Coomassie Brilliant Blue method was used to measure the amount of soluble protein (Hou et al., 2007). A centrifuge tube was filled with 0.2 g of fresh leaves and 50 mM phosphate buffer. Following grinding with a high-throughput tissue grinder, the homogenate was centrifuged at 10,000×g. Using a spectrophotometer, the absorbance was measured at 595 nm after the mixture containing supernatant, distilled water, and Coomassie Brilliant Blue was left to stand for 5 min.

The anthrone colorimetric method was used to measure soluble sugar content (Wang et al., 2024). Using a centrifuge tube, 0.05 g of dry leaves was weighed and extracted twice at 80°C using 80% ethanol (v/v). The supernatant was collected, the supernatants mixed, a small quantity of activated carbon was added, decolorized for 30 min at 80°C, and 10 mL of deionized water was added. After filtering to extract the filtrate, 250 µL of the filtrate was mixed in 5 mL of anthracene solution and placed in a deionized water bath at 100°C for 10 min. Using a spectrophotometer, the absorbance was measured at 625 nm.

The glutathione (GSH) level was measured using the 5,5-dithiobis-(2-nitrobenzoic acid) (DTNB) method (Anderson, 1985), which involved weighing 0.2 g of fresh leaves and 10% C_2_HCl_3_O_2_ in a centrifuge tube, grinding the leaves to homogenize them, and centrifuging at 10,000×g. Finally, following 10 min at 30°C, the absorbance was determined at 412 nm in the mixture of NaHPO_4_, DTNB, and supernatant by utilizing a spectrophotometer.

#### 2.3.4 Determination of active ingredients of plant roots

The components of *S. miltiorrhiza* were determined by high-performance liquid chromatography, with acetonitrile as the mobile phase A and 0.05% phosphoric acid solution as the mobile phase B. The flow rate was stable at 1 mL/min, the column temperature was 30°C, and the detection wavelength was 286 nm. The gradient elution conditions for the fat-soluble components were 0–6 min (mobile phase A was stable at 61%, B was stable at 39%), 6–20 min (mobile phase A increased from 61% to 90%, B decreased from 39% to 10%), 20–20.5 min (mobile phase A decreased from 90% to 61%, B increased from 10% to 39%), and 20.5–25 min (mobile phase A was stable at 61%, B was stable at 39%). The elution conditions of water-soluble components were as follows: 0–15 min (mobile phase A increased from 17% to 23%, B decreased from 83% to 77%), 15–30 min (mobile phase A rise from 23% to 25%, B reduce from 77% to 75%), 30–40 min (mobile phase A rise from 25% to 90%, B decreased from 77% to 10%), and 20.5–25 min four-stage gradient elution (mobile phase A was stabilized at 90%, B was stabilized at 10%).

#### 2.3.5 Determination of soil factors and Cd content

Organic carbon (SOM) was determined using the scorching mass method, where soil samples were dried at 80°C for 1 h and subsequently burned in a muffle furnace for 4 h (Heiri et al., 2001). Soil alkaline phosphatase was assessed using the p-nitrophenyl phosphate method (Tarafdar and Marschner, 1994). Urease activity (U) was assessed using a modified colorimetric method (Hoffmann and Teicher, 1961). The colorimetric method using 3,5-dinitrosalicylic acid was used to determine sucrose (Li et al., 2018). The sodium bicarbonate leaching-molybdenum antimony colorimetric method was used to measure the amount of available phosphorus (Gong et al., 2024). The alkaline-dissolved diffusion method (He et al., 2022) was used to evaluate available nitrogen, and the leaching turbidimetric method (Tian et al., 2021) was used to determine available potassium.

Plant Cd content was determined by oven-drying the harvested aboveground parts and the roots for 48 h at 65°C. Dried samples (approximately 0.2 g) were digested at 200°C–250°C using a mixed HNO_3_/HClO_4_ (3:1) (v/v) digestion solution. Finally, the clarified solution was fixed to 50 mL with 0.2% HNO_3_. Next, ICP-OES was used to measure the amount of Cd present in various plant sections.

For the chemical form of Cd content in soil, the successive extraction method was used (Tessier et al., 1979). The exchangeable form (EXC-Cd), carbonate-bound form (CAB-Cd), iron-manganese oxide-bound form (FMO-Cd), organic matter-bound form (OM-Cd), and residual form (RES-Cd) of Cd were the five operationally identifiable chemical fractions, with 1 M MgCl_2_, 1 M CH_3_COONa, 0.04 M NH_2_OH-HCl, and 0.02 M HNO_3_+30% H_2_O_2_ used to extract the four forms from the soil, respectively.

For RES Cd determination, the remaining soil samples were digested at 190°C using a fully automated microwave digestion instrument (ZM-MID12, Shanghai Zhuoguang Instrument Technology Co., Ltd.) by placing the remaining soil samples in a digestion tube and adding a mixture of HF: HCL: HNO_3_ (1:1:2, v: v: v). Finally, the concentration of Cd in different forms was measured using ICP-OES.

### 2.4 Statistical analyses

Using IBM SPSS Statistics 25, one-way analysis of variance (ANOVA) was conducted to evaluate the influence of Cd stress on multiple indices of PS-2 strains, such as biomass, melanin, malondialdehyde, Cd content, soluble sugar, and soluble protein content. Duncan’s multiple-range tests were utilized to determine the significance of the results between the two groups (*P <0.05*). The effects of DSE inoculation, Cd stress, and their interactions on *S. miltiorrhiza* morphological traits, biomass, and physiological and medicinal components, and Cd content and soil factors were analyzed by two-way ANOVA. The effect size was determined using the partial ETA squared (η_p2_). The influence of Cd stress and DSE on various performance and stress tolerance indices of *S. miltiorrhiza*, such as morphological traits, biomass, physiological traits, active ingredients, soil factors, and Cd content, were evaluated using one-way analysis of variance (ANOVA) and independent samples t-test in IBM SPSS Statistics 25. The significance of the results of the two groups was examined using Duncan’s multiple-range test (*P <0.05*). Excel 2021 was used for data processing and chart creation, and Origin 2022 was used to create graphs and histograms. Variance partitioning analysis (VPA) was performed to examine the effects of DSE and Cd as single components on *S. miltiorrhiza* biomass, morphological and physiological indicators, active ingredients, soil factors, and plant Cd concentration using RStudio version 4.1.1 software.

## 3 Results

### 3.1 Cd-contaminated environment tolerance of DSE *in vitro*

**Figure 1:**
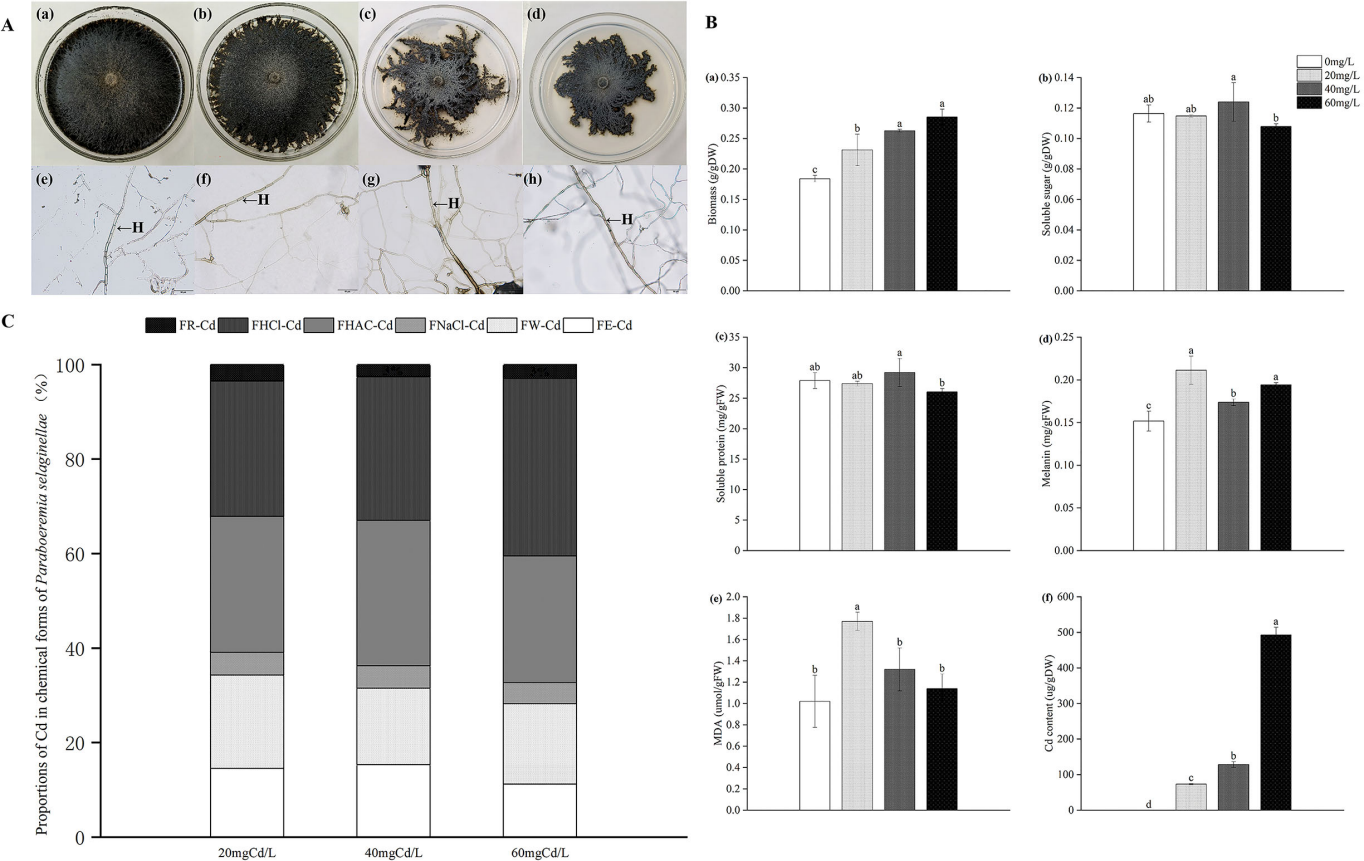
The colony morphology and denoted mycelial morphology of *P. selaginellae* under different levels of Cd stress **(A)**, H: DSE mycelium, bars = 50 µm. Impacts of Cd stress on *P. selaginellae* biomass and physiological indicators **(B)**. The effects of Cd stress were tested by one-way analysis of variance (ANOVA). Different lowercase letters above the error bars indicate a significant difference at *P < 0.05* by Duncan’s multiple-range test. The values reported in the figures are the means of at least three replicates. FW indicates fresh weight and DW indicates dry weight. The impacts of different Cd concentrations on chemical forms of Cd in *P. selaginellae* strain **(C)**. FE, FW, FNaCl, FHAc, FHCl, and FR represent the proportion of Cd in 80% ethanol, deionized water, 1 M NaCl, 2% acetic acid, 0.6 M HCl, and the residual, respectively. The Cd proportion of each chemical form is equal to the Cd content in each chemical form divided by the total Cd content in tissues.

With the increase in Cd stress, the mycelium color became darker, and the colony area decreased significantly when the concentration of Cd was 60 mg Cd/L (**Figure 1A**).

With rising Cd stress levels, strain PS-2 biomass tended to increase and peaked at 60 mg Cd/L (**Figure 1B**-a). Compared with 0 mg Cd/L, the biomass of strain PS-2 increased by 25.73%, 42.75%, and 55.03% at 20, 40, and 60 mg Cd/L, respectively. At 40 mg Cd/L, the content of soluble sugar and soluble protein contents of strain PS-2 increased (**Figure 1B**-b, c). The melanin and malondialdehyde contents of strain PS-2 increased and then decreased with an increase in Cd stress level (**Figures 1B**-d, e), and the melanin and malondialdehyde contents reached the highest level at 20 mg Cd/L, increasing by 39.34% and 74.29%, respectively. The Cd content of strain PS-2 under Cd stress rose gradually with increasing Cd concentration (**Figure 1B**-f), and the Cd content reached 73.23, 128.57, and 492.83 µg/g at 20, 40, and 60 mg Cd/L, respectively. Most of the Cd in strain PS-2 existed in the form of FHCl-Cd (29%–38%), followed by FHAC-Cd (27%–31%), FW-Cd (16%–20%), FE-Cd (11%–15%), FNaCl-Cd (4%–5%), and FR-Cd (3%–4%). As Cd stress increased, the percentage of FHCl-Cd relative to total Cd reached a maximum of 38% at 60 mg Cd/L, while the percentage of FE-Cd relative to total Cd decreased to a minimum of 11% (**Figure 1C**).

### 3.2 DSE colonization rate

**Figure 2:**
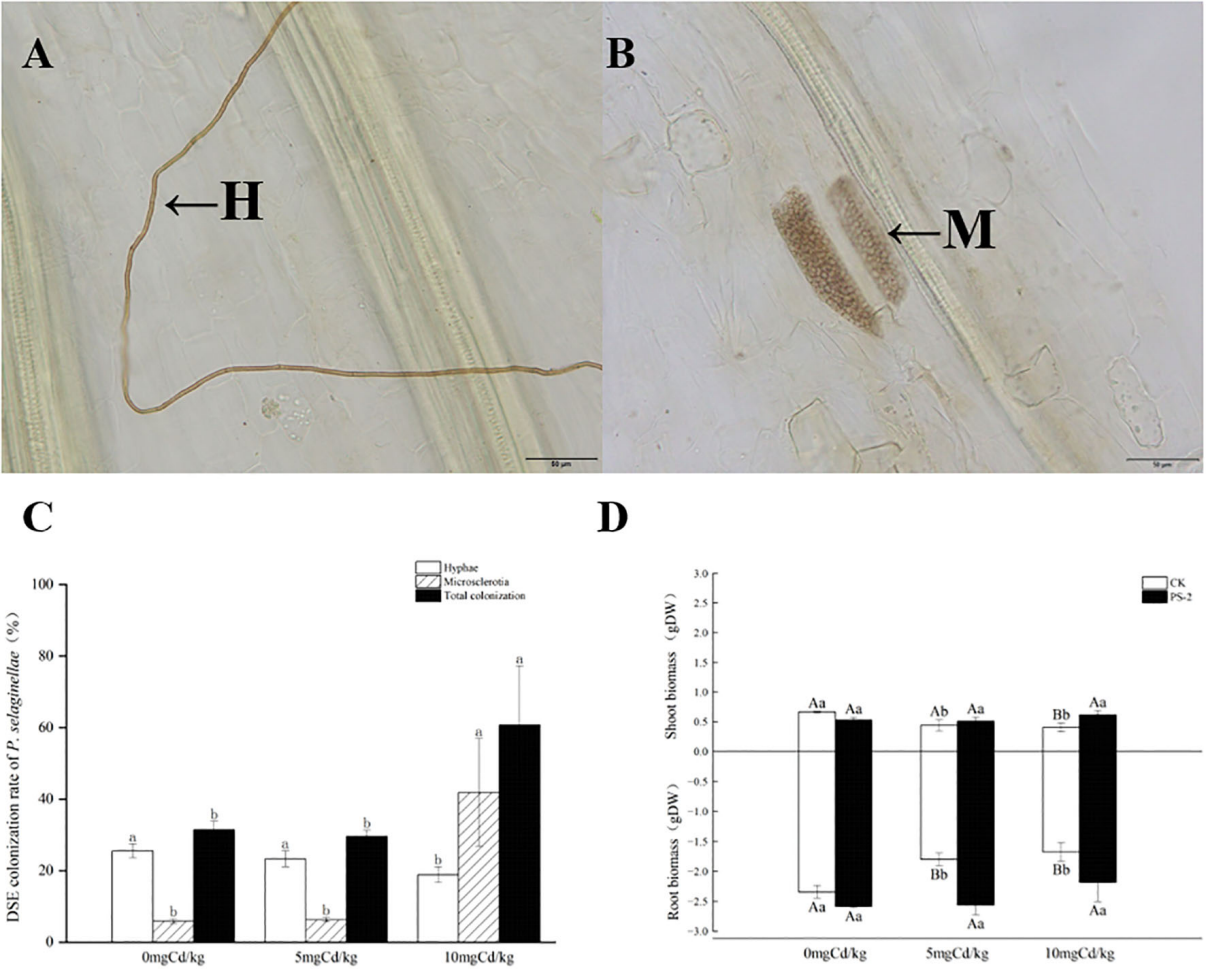
The colonization structures of *P. selaginellae* strains in the roots of inoculated *S. miltiorrhiza* **(A, B)**, H: DSE mycelium, M: DSE microsclerotium. Bars = 50 µm. *P. selaginellae* colonization rate in inoculated *S. miltiorrhiza* roots **(C)**, and impacts of Cd-contaminated soil and *P. selaginellae* inoculation on plant biomass **(D)**. CK: non-inoculated control, PS-2: inoculated *P. selaginellae.* FW indicates fresh weight and DW indicates dry weight. The effects of Cd stress and DSE inoculation were tested by one-way analysis of variance (ANOVA) and t-test. Different capital letters above the error bars indicate significant differences between uninoculated and inoculated DSE treatments in the same Cd-contaminated soil at *P < 0.05* by Duncan’s multiple-range test; different lowercase letters indicate significant differences between different Cd-contaminated soils for the same inoculated treatment. The values reported in the figures are the means of at least three replicates.

Following harvesting, the PS-2 inoculated roots exhibited typical septate hyphae and microsclerotia structures (**Figures 2A, B**).

The colonization of DSE under different treatments was measured, with mycelial colonization ranging from 18.9% to 25.6%, microsclerotium colonization ranging from 5.9% to 41.85%, and total root colonization ranging from 31.5% to 60.7%. Cd-contaminated soil significantly increased the colonization of PS-2. Microsclerotium and total colonization levels reached a maximum at 10 mg Cd/kg soil (**Figure 2C**).

### 3.3 Morphological traits and biomass of *S. miltiorrhiza* plants

DSE inoculation with Cd-contaminated soil and the interaction of the two factors had significant effects on biomass above and below ground. The belowground biomass was significantly mainly affected by DSE (*P<0.001*, η_p2_ = 0.761) (**Table 1**). The aboveground biomass at 5 mg Cd/kg and 10 mg Cd/kg soil rose by 16.81% and 52.33%, respectively, following DSE inoculation in comparison to the control. DSE inoculation increased subterranean biomass for the 0, 5, and 10 mg Cd/kg soil conditions by 10.21%, 42.35%, and 30.21%, respectively, in comparison to the non-inoculated control (**Figure 2D**).

**Table 1:** Two-way analysis of variance of the influence of DSE inoculation and Cd contamination on the growth, physiological and medicinal components, Cd content, and soil factors of *S. miltiorrhiza*.

	DSE			Cd-contaminated soil			DSE * Cd-contaminated soil		
	*F*	*P*	η_p2_	*F*	*P*	η_p2_	*F*	*P*	η_p2_
Leaf number	0.211	0.655	0.017	1.434	0.276	0.193	0.645	0.542	0.097
Plant height	12.702	**< 0.01**	0.514	2.222	0.151	0.27	0.567	0.582	0.086
Root length	27.37	**<0.001**	0.695	0.843	0.454	0.123	2.193	0.154	0.268
Root surface area	14.676	**< 0.01**	0.55	6.295	**< 0.05**	0.512	3.075	0.084	0.339
Root diameter	1.267	0.282	0.095	1.983	0.18	0.248	1.643	0.234	0.215
Root volume	5.369	**< 0.05**	0.309	2.045	0.172	0.254	8.053	**< 0.01**	0.573
Shoot biomass	2.701	0.126	0.184	5.587	**< 0.05**	0.482	10.53	**< 0.01**	0.637
Root biomass	38.219	**<0.001**	0.761	14.347	**< 0.01**	0.705	3.447	0.066	0.365
Glutathione	16.75	**< 0.001**	0.583	17.165	**< 0.001**	0.741	8.317	**< 0.01**	0.581
Soluble protein	4.563	0.054	0.275	2.821	0.099	0.32	0.98	0.403	0.14
Soluble sugar	75.152	**< 0.001**	0.862	8.235	**< 0.05**	0.578	35.432	**< 0.001**	0.855
Chlorophyll	34.274	**< 0.001**	0.741	2.38	0.135	0.284	7.341	**< 0.01**	0.55
Tanshinone I	0.458	0.511	0.037	2.31	0.142	0.278	14.924	**< 0.01**	0.713
Tanshinone IIA	0.23	0.64	0.019	2.092	0.166	0.259	3.212	0.076	0.349
Cryptotanshinone	25.255	**< 0.001**	0.678	0.669	0.53	0.1	8.376	**< 0.01**	0.583
Salvianolic acid B	1.282	0.28	0.097	8.335	**< 0.01**	0.581	37.914	**< 0.001**	0.863
Available nitrogen	80	**< 0.001**	0.87	2.6	0.115	0.302	5	**< 0.05**	0.455
Available phosphorus	5.725	**< 0.05**	0.323	0.156	0.857	0.025	4.002	**< 0.05**	0.4
Available potassium	17.381	**< 0.01**	0.592	0.822	0.463	0.12	1.365	0.292	0.185
Organic carbon	30.22	**< 0.001**	0.716	3.716	0.055	0.382	1.577	0.247	0.208
Invertase	1.954	0.187	0.14	7.262	**< 0.01**	0.548	1.681	0.227	0.219
Urease	25.942	**< 0.01**	0.684	2.057	0.171	0.255	4.205	**< 0.05**	0.412
Alkaline phosphatase	2.127	0.17	0.151	12.042	**< 0.001**	0.667	0.222	0.804	0.036
Shoot Cd	247.733	**< 0.001**	0.954	12197.37	**< 0.001**	1	90.432	**< 0.001**	0.938
Root Cd	32.041	**< 0.001**	0.728	31422.69	**< 0.001**	1	42.938	**< 0.001**	0.877

DSE indicates different inoculum (control and *P. selaginellae*); Cd-contaminated soil indicates different Cd contamination levels (0, 5, and 10 mg Cd/kg soil); the interaction effect of DSE and Cd-contaminated soil is indicated by DSE * Cd-contaminated soil; significant *P*-values are in bold. The partial ETA square (η_p2_) indicates the effect size of different factors.

**Figure 3:**
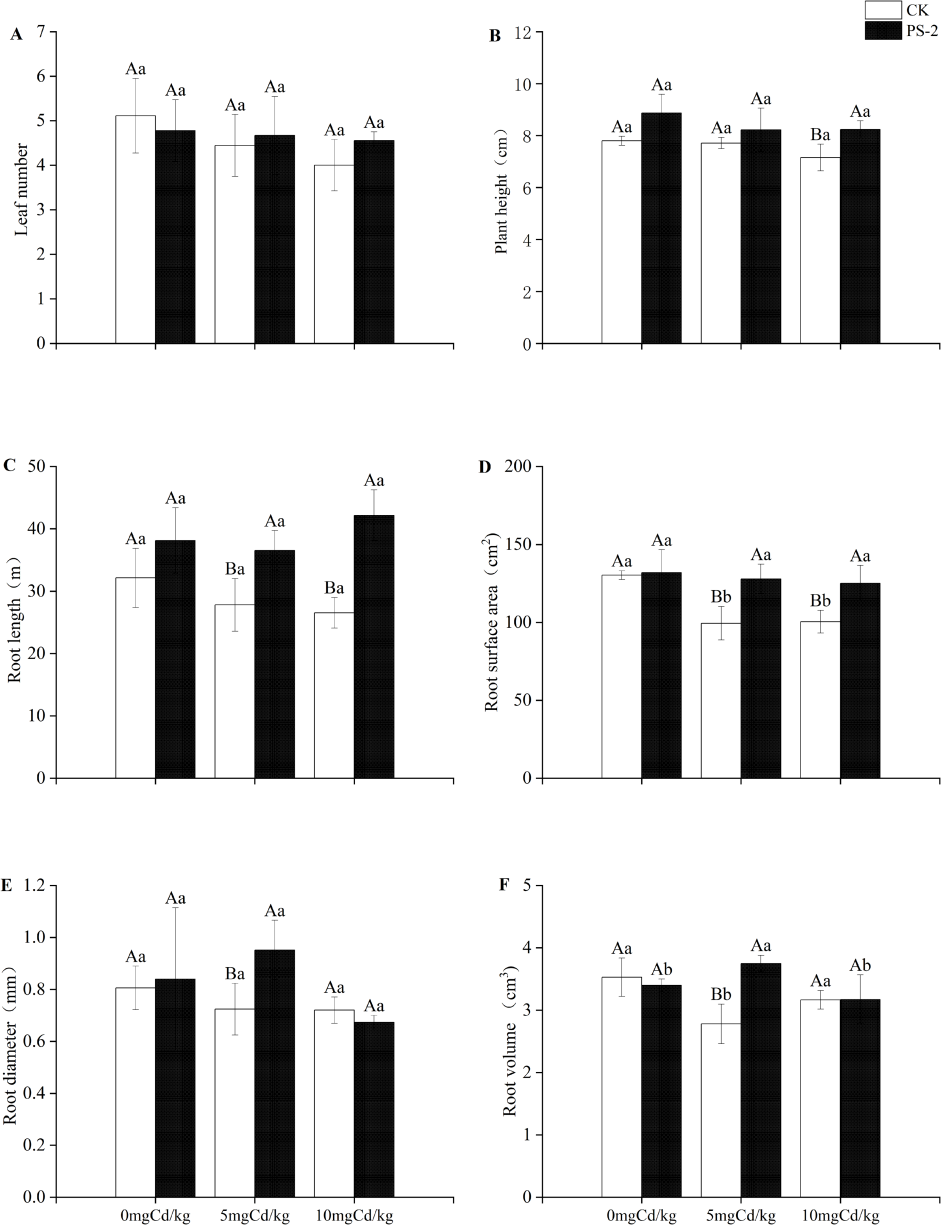
Impacts of *P. selaginellae* inoculation and Cd-contaminated soil on *S. miltiorrhiza* morphological indices. **(A)** Leaf number, **(B)** Plant height, **(C)** Root length, **(D)** Root surface area, **(E)** Root diameter, **(F)** Root volume. CK: non-inoculated control, PS-2: inoculated *P. selaginellae.* FW indicates fresh weight and DW indicates dry weight. The effects of Cd stress and DSE inoculation were tested by one-way analysis of variance (ANOVA) and t-test. Different capital letters above the error bars indicate significant differences between uninoculated and inoculated DSE treatments in the same Cd-contaminated soil at *P < 0.05* by Duncan’s multiple-range test; different lowercase letters indicate significant differences between different Cd-contaminated soils for the same inoculated treatment. The values reported in the figures are the means of at least three replicates.

DSE inoculation with Cd-contaminated soil and the interaction of the two factors had significant effects on plant morphology above and below ground. The root length was significantly mainly affected by DSE (*P < 0.001*, ηp² = 0.695). (**Table 1**). Plant morphology was less negatively influenced by Cd-contaminated soil after DSE inoculation. When compared to the uninoculated treatment, the DSE inoculation increased plant height by 13.68%, root length by 18.70%, root surface area by 1.29%, and root diameter by 4.22% in the 0 mg Cd/kg soil (**Figures 3B-E**). DSE inoculation increased plant leaf number 5.00%, plant height 6.63%, plant root surface area 31.35%, plant root diameter 28.61%, plant root volume by 31.42%, and plant root length by 34.89% in the 5 mg Cd/kg soil, compared with the uninoculated treatment (**Figures 3A–F**). When compared to the uninoculated treatment, the DSE inoculation increased leaf number by 13.89%, plant height by 15.06%, root length by 58.91%, and root surface area by 24.54% in the 10 mg Cd/kg soil (**Figures 3A–D**).

### 3.4 Physiological and medicinal components and Cd content of *S. miltiorrhiza*

**Figure 4:**
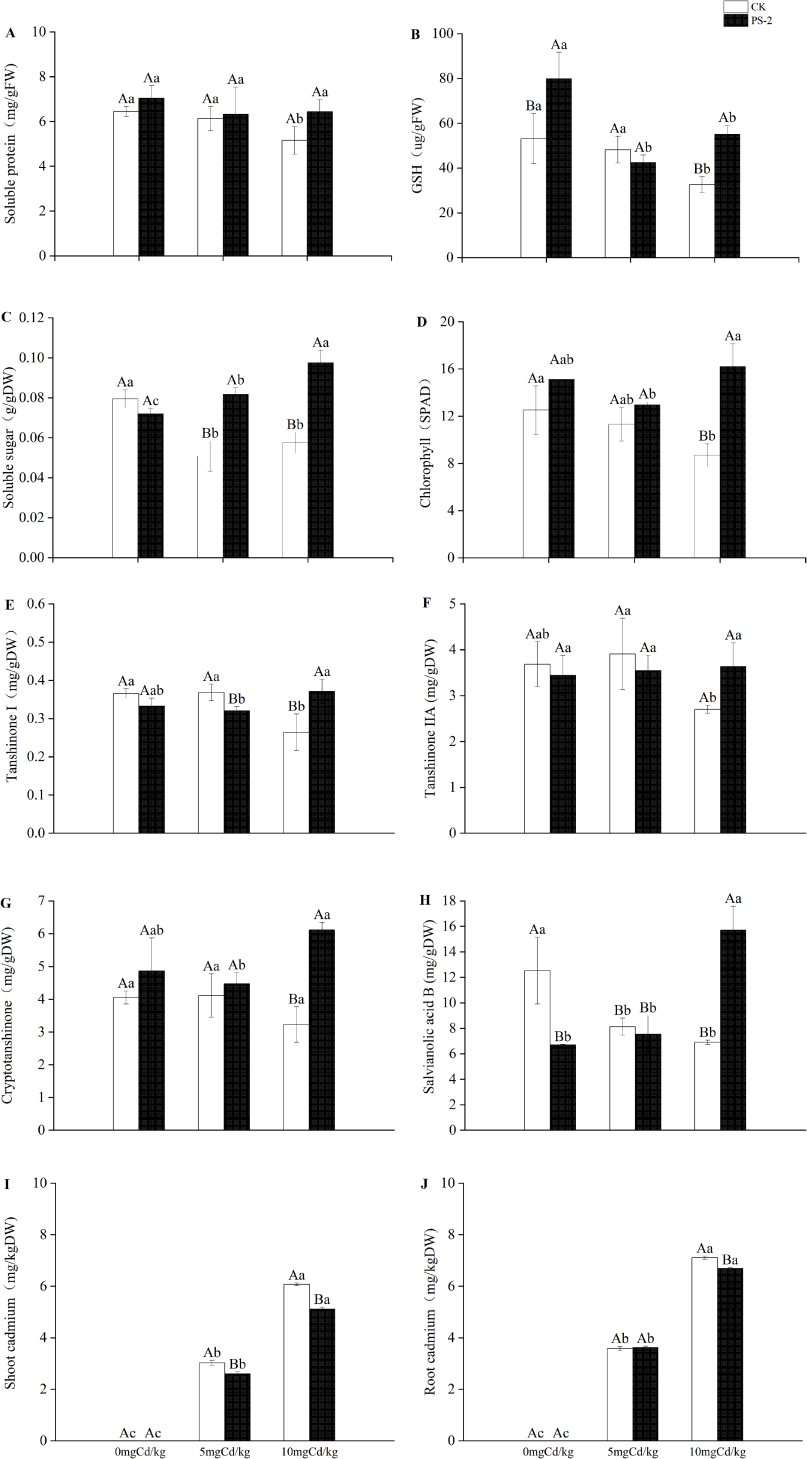
Impacts of *P. selaginellae* inoculation and Cd-contaminated soil on *S. miltiorrhiza* physiological indices **(A-D)**, content of medicinal components **(E-H)**, and plant Cd content **(I, J)**. CK: non-inoculated control, PS-2: inoculated *P. selaginellae.* FW indicates fresh weight and DW indicates dry weight. The effects of Cd stress and DSE inoculation were tested by one-way analysis of variance (ANOVA) and t-test. Different capital letters above the error bars indicate significant differences between uninoculated and inoculated DSE treatments in the same Cd-contaminated soil at *P < 0.05* by Duncan’s multiple-range test; different lowercase letters indicate significant differences between different Cd-contaminated soils for the same inoculated treatment. The values reported in the figures are the means of at least three replicates.

DSE inoculation with Cd-contaminated soil and the interaction of the two factors had significant effects on plant levels of physiological components. Chlorophyll, soluble sugar, and glutathione were significantly mainly affected by DSE (*P < 0.001*, ηp² = 0.741; *P < 0.001*, ηp² = 0.862; *P < 0.001*, ηp² = 0.583) (**Table 1**). The DSE-inoculated treatment increased the levels of soluble protein by 9.08%, glutathione by 50.44%, and chlorophyll by 20.76% in the 0 mg Cd/kg soil, compared with non-inoculated control (**Figures 4A, B, D**). The DSE-inoculated treatment increased the levels of soluble protein by 3.20%, soluble sugar by 60.74%, and chlorophyll by 14.52% in the 5 mg Cd/kg soil, compared with the non-inoculated control (**Figures 4A, C, D**). The DSE-inoculated treatment increased the amounts of soluble protein by 24.99%, glutathione by 68.86%, soluble sugar by 69.53%, and chlorophyll by 85.97% in the 10 mg Cd/kg soil, compared with the non-inoculated control (**Figures 4A–D**).

DSE inoculation with Cd-contaminated soil and the interaction of the two factors had significant effects on medicinal components (**Table 1**). When compared with the non-inoculated control, the DSE inoculation increased the concentration of cryptotanshinone in the soil treatments of 0 and 5 mg Cd/kg by 19.95% and 8.74%, respectively (**Figure 4G**). When compared with the non-inoculated control, inoculation with DSE increased the levels of tanshinone I by 40.65%, tanshinone IIA by 34.51%, cryptotanshinone by 89.42%, and salvianolic acid B by 127.45% in the 10 mg Cd/kg soil (**Figures 4E–H**).

DSE inoculation with Cd-contaminated soil and the interaction of the two factors had significant effects on the aboveground and belowground Cd of *S. miltiorrhiza* (**Table 1**). When compared to the uninoculated treatment, the DSE inoculation decreased the amount of Cd accumulation in the aboveground portion of the plants by 13.91% in the 5 mg/kg soil (**Figure 4I**). When compared to the uninoculated treatment, PS-2 inoculation decreased the amount of Cd that accumulated in the aboveground and underground parts of the plants by 15.82% and 5.92%, respectively, at a soil concentration of 10 mg/kg (**Figures 4I, J**).

### 3.5 Soil factor

**Figure 5:**
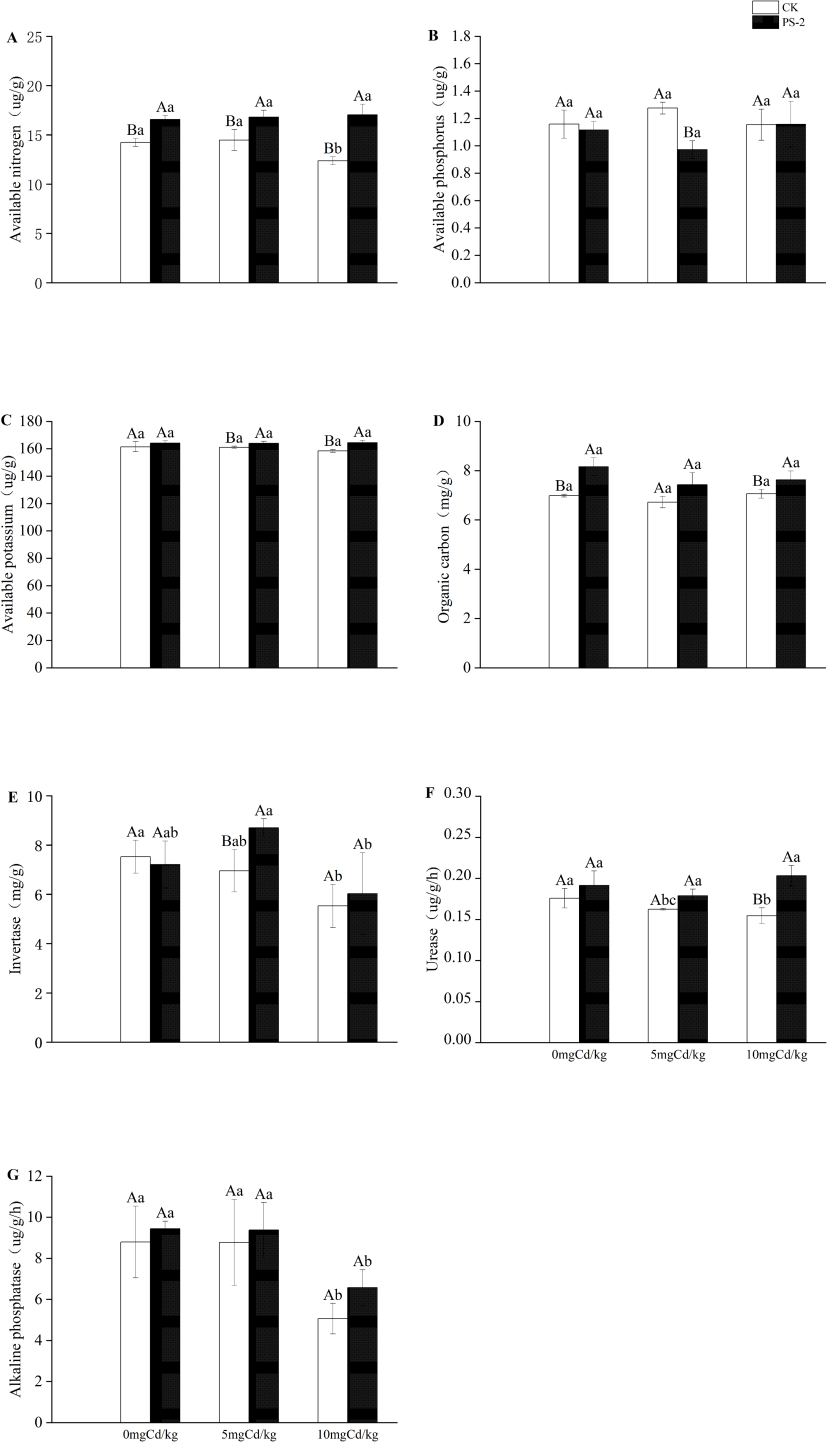
Impacts of *P. selaginellae* inoculation and Cd-contaminated soil on soil factors. **(A)** Available nitrogen, **(B)** Available phosphorus, **(C)** Available potassium, **(D)** Organic Carbon, **(E)** Invertase, **(F)** Urease, **(G)** Alkaline phosphatase. CK: non-inoculated control, PS-2: inoculated *P. selaginellae.* The effects of Cd stress and DSE inoculation were tested by one-way analysis of variance (ANOVA) and t-test. Different capital letters above the error bars indicate significant differences between uninoculated and inoculated DSE treatments in the same Cd-contaminated soil at *P < 0.05* by Duncan’s multiple-range test; different lowercase letters indicate significant differences between different Cd-contaminated soils for the same inoculated treatment. The values reported in the figures are the means of at least three replicates.

DSE inoculation with Cd-contaminated soil and the interaction of the two factors had significant effects on soil factors. Available nitrogen and organic carbon were significantly mainly affected by DSE. (*P < 0.001*, ηp² = 0.87; *P < 0.001*, ηp² = 0.716) (**Table 1**). The DSE-inoculated treatment increased the contents of soil available nitrogen by 16.39%, available potassium by 1.63%, organic carbon by 16.86%, urease by 9.00%, and alkaline phosphatase by 7.32% in the 0 mg Cd/kg soil, compared with the uninoculated treatment (**Figures 5A, C, D, F, G**). The DSE-inoculated treatment increased the contents of soil available nitrogen by 16.13%, available potassium by 1.77%, organic carbon by 10.51%, sucrase by 25.10%, urease by 10.13%, and alkaline phosphatase by 6.84% in the 5 mg Cd/kg soil, compared with the uninoculated treatment (**Figures 5A, C-G**). The PS-2 inoculated treatment increased the contents of soil effective nitrogen by 37.74%, available phosphorus by 0.29%, available potassium by 3.75%, organic carbon by 7.93%, sucrase by 9.06%, urease by 31.55% and alkaline phosphatase by 29.82% in the 10 mg Cd/kg soil, compared with the uninoculated treatment (**Figures 5A–G**).

### 3.6 Chemical form of soil Cd

**Figure 6:**
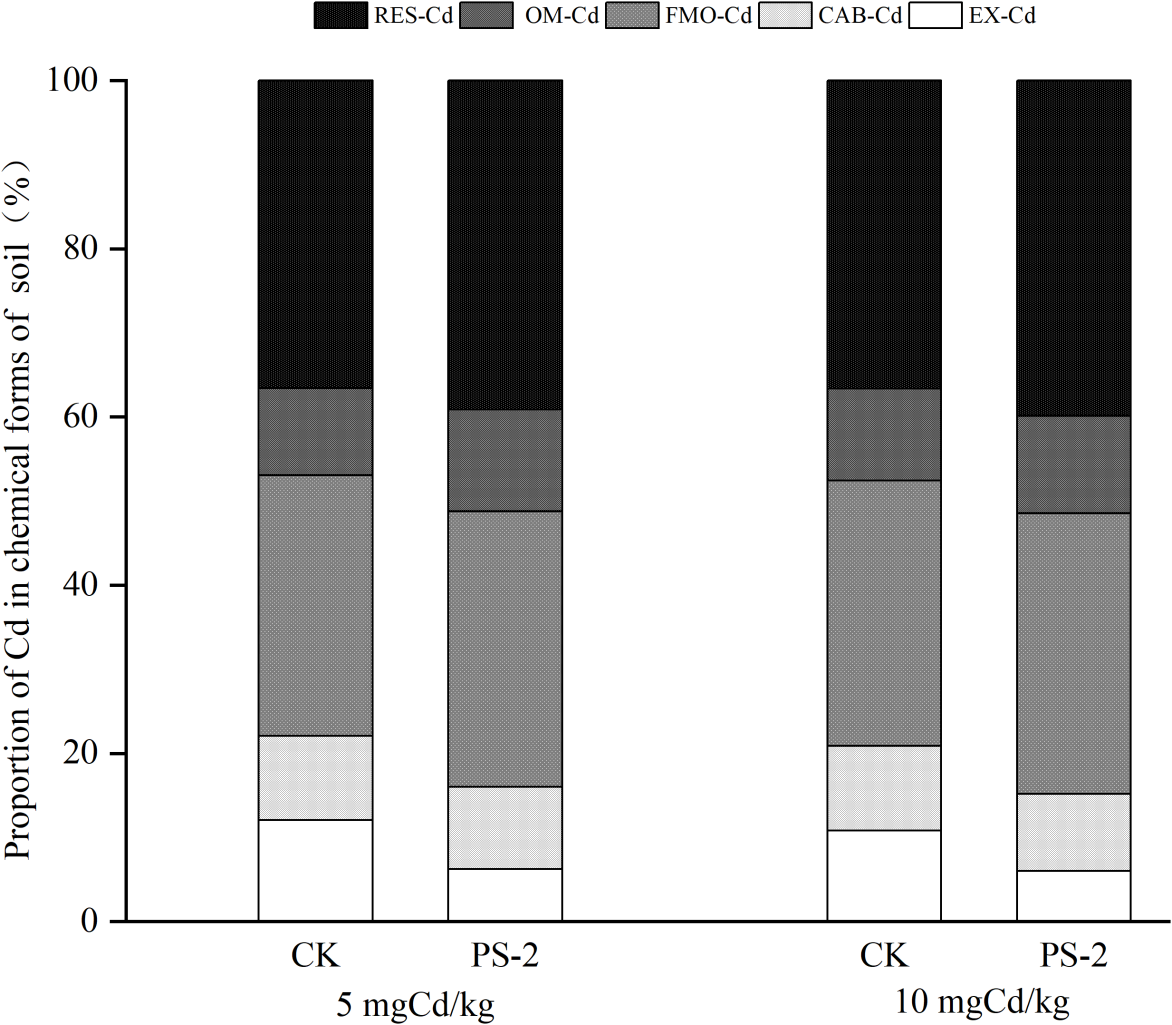
Impacts of different Cd-contaminated soils and *P. selaginellae* inoculation on the chemical forms of Cd in soil. EX, CAB, FMO, OM, and RES represent the proportion of Cd in exchangeable form, carbonate-bound form, iron-manganese oxide-bound form, organic matter-bound form, and residual form, respectively. The Cd proportion of each chemical form is equal to the Cd content in each chemical form divided by the total Cd content in tissues.

The chemical form of soil Cd was dominated by RES-Cd (37%–40%), followed by FMO-Cd (31%–33%), OM-Cd (10%–12%), EX-Cd (6%–12%), and CAB-Cd (9%–10%). In the 5 mg Cd/kg soil, PS-2 inoculation decreased EX-Cd and CAB-Cd by 27.27% and increased OM-Cd and RES-Cd by 8.51% as compared to the non-inoculated control. In the 10 mg Cd/kg soil, PS-2 inoculation decreased EX and CAB-Cd by 28.57% as compared to the uninoculated treatment and increased OM and RES-Cd by 8.33% (**Figure 6**).

### 3.7 Variation partitioning analyses

**Figure 7:**
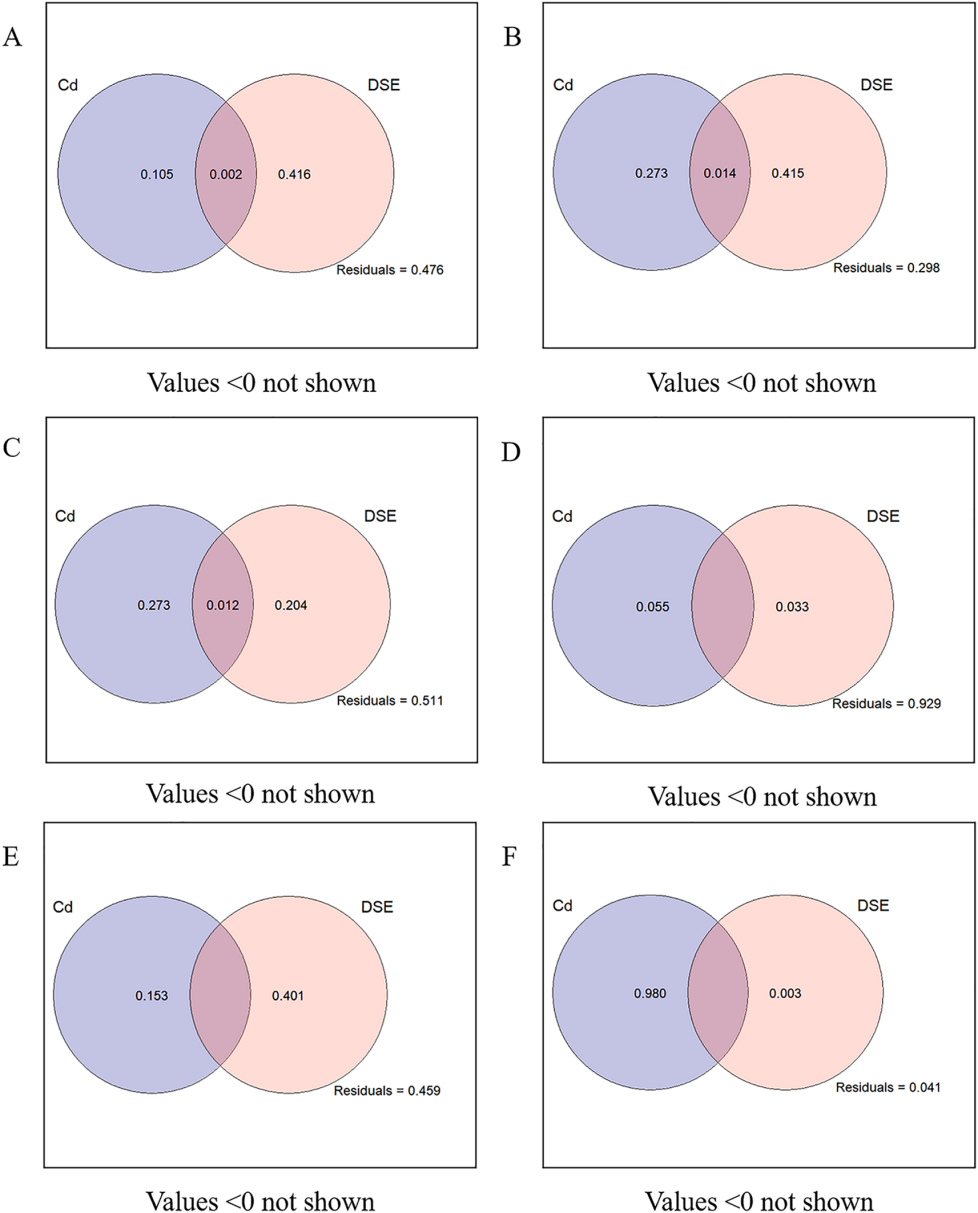
VPA analysis of *P. selaginellae* inoculation and different Cd-contaminated soils on the growth and physiological indices, soil factors, and plant Cd content of *S. miltiorrhiza*. **(A-F)** The impacts of *P. selaginellae* inoculation and different Cd-contaminated soils on the morphology, biomass, physiology, medicinal components, soil factors, and plant Cd content of *S. miltiorrhiza*, respectively.

VPA was used to estimate the influence of Cd-contaminated soil and DSE inoculation on plant physiology, growth indexes, soil factors, and plant Cd content of *S. miltiorrhiza* (**Figure 7**). Cd-contaminated soil and DSE inoculation independently explained 10.5% and 41.6% of the growth morphology of *S. miltiorrhiza*, and the common explanation was 0.2%. DSE inoculation had a relatively large effect on the growth of *S. miltiorrhiza* (**Figure 7A**). Cd-contaminated soil and DSE inoculation independently explained 27.3% and 41.5% of the biomass of *S. miltiorrhiza*, respectively, with a common explanation of 1.4%. DSE inoculation had a relatively large effect on the biomass of *S. miltiorrhiza* (**Figure 7B**). Cd-contaminated soil and DSE species explained 27.3% and 20.4% of the physiological indices of *S. miltiorrhiza*, respectively, while the combined explanation was 1.2%, indicating that Cd-contaminated soil had a relatively greater influence on the physiological indices of *S. miltiorrhiza* (**Figure 7C**). Cd-contaminated soil and DSE inoculation independently explained 5.5% and 3.3% of the medicinal components of *S. miltiorrhiza*, and the influence of Cd-contaminated soil on the medicinal components was relatively large (**Figure 7D**). Cd-contaminated soil and DSE species separately explained 15.3% and 40.1% of the soil factors, respectively, and DSE inoculation had a relatively large effect on the soil factors (**Figure 7E**). The Cd-contaminated soil and DSE inoculation separately explained 98% and 0.3% of the Cd content, and the influence of Cd-contaminated soil on the Cd content in *S. miltiorrhiza* was relatively large (**Figure 7F**).

## 4 Discussion

One of the most challenging environmental issues currently is soil Cd pollution, particularly when it comes to agricultural output and the growing of medicinal plants (Zhou et al., 2020). One of the best methods for enhancing plants in stressful conditions is the combination of plants and endophytic fungus (Cozzolino et al., 2016; Zhu et al., 2021). DSEs have a high tolerance to HM, are widely dispersed in heavy metal-contaminated habitats, and have been demonstrated to increase host plant adaptability to an HM-contaminated environment (Su et al., 2021).

### 4.1 DSE tolerance to Cd-contaminated environment

The present study indicated that although a Cd-contaminated environment had a negative impact on the growth of *P. selaginellae*, this strain still exhibited sustained activity even at a concentration of 60 mg Cd/L. Meanwhile, the biomass of *P. selaginellae* exceeded the control group at 0, 20, 40, and 60mg Cd/L, and mycelial Cd content had a significant increase with increasing levels of Cd contamination, indicating that *P. selaginellae* has a high tolerance to Cd stress. Typically, organisms are severely influenced by heavy metal accumulation, which results in oxidative damage to cells (Mansoor et al., 2023). To ascertain the influence of a Cd-contaminated environment on antioxidant substances, the MDA, soluble protein, soluble sugar, and melanin levels in the DSE strains were assessed in the current investigation. The degree of damage caused by oxidative stress can be determined using MDA, a biomarker of oxidative stress (Ma et al., 2019). Here, the accumulation of MDA in *P. selaginellae* at 20–60 mg Cd/L demonstrated that Cd stress had a negative influence on the DSE strains.

Important osmoregulatory compounds that help organisms become more resistant to dehydration include soluble proteins and soluble carbohydrates (Qing et al., 2018). *P. selaginellae* accumulation of soluble protein and soluble sugar counterbalanced the solute potential, reducing the detrimental effects of Cd contamination and promoting cell development. It has been demonstrated that melanin, a major component of the fungal cell wall of DSEs, increases the species’ ability to survive in harsh environments (Potisek et al., 2021). Some studies have shown that melanin is not related to the tolerance of DSE strains to extreme environments (Gaber et al., 2020; Berthelot et al., 2020). However, some studies have shown that increased melanin concentration may help them resist heavy metal stress (Cao et al., 2023; Hou et al., 2020). In our study, compared with the control, the melanin content of *P. selaginellae* increased under all cadmium stress gradients, which may be the reason why *P. selaginellae* has stronger cadmium resistance than other DSEs.

Furthermore, we noticed a notable alteration in the chemical forms of Cd in *P. selaginellae.* The chemical form of Cd has a significant influence on its reactivity and solubility, which in turn influence how biotoxic it is to cells. Among the various chemical forms of Cd, cadmium phosphate complexes extracted by 2% acetic acid (FHAc-Cd) and by 1 M NaCl (FHCl-Cd), and FR-Cd are important for organisms’ tolerance due to the insolubility, low mobility, and low toxicity of cadmium (Lu et al., 2024). In this study, the *P. selaginellae* strain accounted for the largest proportion of low toxicity FHCl-Cd and FR-Cd, which accounted for 33%–41% of total Cd content, which was similar to a previous study (Huang et al., 2019), where the low toxicity FHCl-Cd content gradually increased and the high toxicity FE-Cd and FW-Cd content gradually decreased with the increase in Cd contamination. This suggested that *P. selaginellae* may alleviate the toxicity of a Cd-contaminated environment by converting Cd from highly toxic FE-Cd and FW-Cd to low toxic FHCl-Cd (Zheng et al., 2022).

### 4.2 Effects of DSE inoculation on the performance of *S. miltiorrhiza* in Cd-contaminated soils

In order to adapt to a heavy metal-contaminated environment, it has been found that plants recruit microorganisms to colonize their roots, which typically have strong heavy metal tolerance, for instance, DSE strains have been isolated from *Salix caprea* and poplars, respectively (Likar and Regvar, 2013; Yung et al., 2021). However, little is known about whether DSE can promote the heavy metal tolerance of medicinal plants. In our study, the total colonization rate of DSE gradually increased with the increase in Cd concentration. Of particular importance was that when Cd content reaches 10 mg/kg soil, the microsclerotia colonization rate significantly increases compared to the no-stress treatment. As an important strategy to resist Cd stress, microsclerotia play a crucial role in the survival of DSE in stressful environments (Santos et al., 2021). Moreover, root length, biomass, volume, and surface area of DSE-inoculated *S. miltiorrhiza* were promoted in Cd-contaminated soil compared to the control plants. When compared to the uninoculated treatment, the DSE inoculation enhanced root length by 18.70% at the 0 mg Cd/kg soil (**Figure 3C**). DSE inoculation increased root surface area by 31.35%, plant root diameter by 28.61%, plant root volume by 31.42%, and plant root length by 34.89% in the 5 mg Cd/kg soil, compared with the uninoculated treatment (**Figures 3C–F**). When compared to the uninoculated treatment, the DSE inoculation increased root length by 58.91% and root surface area by 24.54% in the 10 mg Cd/kg soil (**Figures 3C, D**). This was similar to previous findings (Suksabye et al., 2015; Gaber et al., 2023; Berthelot et al., 2017a). These results indicate that the improvement of plant root morphology enhances water and nutrient utilization efficiency, which may make plants more adaptable to Cd-contaminated soils (Naciri et al., 2021).

Cd stress interferes with photosynthesis mechanisms and negatively affects chlorophyll content, and lower pigment content may be one of the main factors for reduced plant growth (Chen et al., 2023). In this study, we found that inoculation with *P. selaginellae* increases the chlorophyll content in different Cd-contaminated soils compared to uninoculated treatments. It has been shown that DSE colonization can improve plant photosynthesis in Cd-contaminated environments (He et al., 2017). In order to alleviate the adverse influence of abiotic stress, plants increase osmotic potential at the cellular level by synthesizing and accumulating osmotic regulators such as soluble sugars and soluble proteins (Öztürk et al., 2020). Our research has found that inoculation with *P. selaginellae* increased the amount of soluble protein and GSH in Cd-contaminated environments compared to the non-inoculated control, and it also significantly raised the amount of soluble sugar in the 5 and 10 mg Cd/kg soils. According to studies, plants that are stressed release reactive oxygen species (ROS), which harm the structure of plant cells. GSH, on the other hand, can accelerate bioreduction processes, remove ROS in cells, and lessen long-term damage (Zheng et al., 2023).

Studies have shown that Cd stress can lead to a decrease in the active ingredients of medicinal plants (Wu et al., 2023b), while endophytic fungi have a positive influence on the active ingredients of medicinal plants (Yuan et al., 2023). This study found that compared with 0 mg Cd/kg soil, high Cd-contaminated soil (10 mg Cd/kg soil) significantly reduced the content of active ingredients in the non-inoculated control plants, and inoculation with *P. selaginellae* increased the content of fat-soluble and water-soluble medicinal ingredients in plant roots under high Cd stress. These results provided a basis for utilizing DSE resources to promote the growth and quality improvement of *S. miltiorrhiza* in Cd-contaminated soils.

### 4.3 Effects of DSE inoculation on soil properties in Cd-contaminated soils

At present, the effect of inoculation with DSEs on soil enzyme activity in a heavy metal-contaminated environment is unclear. It has been reported that Cd-contaminated environments seriously affect soil physicochemical properties and soil enzyme activity, thereby reducing soil fertility (Huang et al., 2018). According to the results of soil factor determination, compared with the non-inoculated control, *P. selaginellae* inoculation increased the activity of soil sucrase, urease, and soil alkaline phosphatase, as well as the content of available nitrogen, available potassium, and organic matter, further affecting the yield and quality of *S. miltiorrhiza* in our study. Previous studies also found that inoculation with DSE increased biomass and mineral nutrient levels and soil enzyme activities (Deng et al., 2020). Soil sucrase can boost the amount of soluble nutrients in the soil and is closely linked to the breakdown and transformation of soil urea (Wu et al., 2023c). Soil urease is a significant catalyst for the breakdown and transformation of soil urea, and its activity is triggered by the plant’s utilization of nitrogen (Li et al., 2022). Soil alkaline phosphatase is involved in the soil phosphorus metabolic cycle and can catalyze the hydrolysis of organic phosphorus to inorganic forms, thereby increasing the availability of phosphorus in the soil (Guo et al., 2023).

### 4.4 Effects of DSE inoculation on plant Cd content and soil Cd chemical forms in Cd-contaminated soils

Cd is extremely slow to degrade in soil and can only undergo transformation and migration. The stronger its migration ability, the greater its toxicity to living organisms (Yu et al., 2023). The chemical forms of soil Cd are EX, CAB, FMO, OM, and RES-Cd from high to low toxicity to organisms (Jiang et al., 2021). This study found that compared with the control, the inoculation with *P. selaginellae* reduced the proportion of highly toxic FE-Cd in soil and increased the proportion of low-toxic OM-Cd and RES-Cd, indicating that that inoculation with DSEs may be a potential detoxification mechanism that can accelerate the transition of soil Cd from a state of easy migration and high toxicity to organisms to a low toxicity state (Bolan et al., 2003). In addition, inoculation with *P. selaginellae* also reduced the accumulated Cd in *S. miltiorrhiza* shoots under different Cd-contaminated soils and Cd content in *S. miltiorrhiza* roots in the 10 mg Cd/kg soil. Previous studies have shown that under Cd stress, promoting the conversion of RES-Cd in the soil can reduce the biological toxicity of Cd in soil and reduce the absorption and accumulation of Cd in plants (Qin et al., 2021), which is similar to our research results. In summary, inoculation with DSEs not only promoted plant growth and improved Cd tolerance but also improved soil physicochemical properties and enzyme activity, reduced the biological toxicity of Cd, and provided a basis for utilizing DSE resources to promote medicinal plant cultivation and improve soil microenvironment ecology in heavy metal-polluted soils. Based on this, we will further investigate whether DSEs can promote the growth and Cd tolerance of medicinal plants such as *Scutellaria baicalensis* in Cd-contaminated conditions.

## 5 Conclusion

This study reported for the first time that the *P. selaginellae* strain has high tolerance to Cd and improves the performance and adaptability *of S. miltiorrhiza* in Cd-contaminated soil. Cd stress reduced the proportion of highly toxic FE-Cd and FW-Cd and increased the proportion of less toxic FHCl-Cd in *P. selagellae* mycelium. In Cd-polluted soil, inoculation with *P. selaginellae* reduced the proportion of highly toxic FE-Cd, increased the proportion of less toxic OM-Cd and RES-Cd in the soil, and reduced Cd accumulation in the plants, especially in the roots. In addition, *P. selaginellae*-colonized roots had improved root structure, physiological parameters, and soil nutrients, thereby promoting the growth and quality of *S. miltiorrhiza*. These results indicate that DSEs had a positive influence on the performance and Cd tolerance of *S. miltiorrhiza*, and exploitation and utilization of DSEs may be a new method for improving the cultivation of medicinal plants in heavy metal-contaminated soils.

## Data Availability

The raw data supporting the conclusions of this article will be made available by the authors, without undue reservation.
